# Who gets screened and who tests positive? Drug screening among justice-involved youth in a midwestern urban county

**DOI:** 10.1186/s40352-024-00273-w

**Published:** 2024-04-05

**Authors:** Richelle L. Clifton, Ian Carson, Allyson L. Dir, Wanzhu Tu, Tamika C.B. Zapolski, Matthew C. Aalsma

**Affiliations:** 1 Department of Pediatrics, University of Washington School of Medicine, Seattle, WA USA; 2 Department of Psychology, Indiana University Indianapolis, 402 N. Blackford St., LD 124, Indianapolis, IN 46202 USA; 3 Department of Psychiatry, Adolescent Behavioral Health Research Program, Indiana University School of Medicine, Indianapolis, IN USA; 4 Department of Biostatistics, Indiana University School of Medicine, Indianapolis, IN USA; 5 Department of Pediatrics, Section of Adolescent Medicine, Adolescent Behavioral Health Research Program, Indiana University School of Medicine, Indianapolis, IN USA

**Keywords:** Drug screening, Juvenile justice, Juvenile probation

## Abstract

**Background:**

Given high rates of substance use among justice-involved youth, justice systems have attempted to monitor use through drug screening (DS) procedures. However, there is discretion in deciding who is screened for substance use, as not every youth who encounters the system is screened. The aim of the current study was to examine factors associated with selection for and results of oral DS among justice-involved youth assigned to probation to better inform potential DS policy. Electronic court records from 4,668 youth with first-incident records assigned to probation in a midwestern urban county’s juvenile justice system between 2011 and 2016 were included in the analytical sample. Race/ethnicity, gender, age, number of charges and charge type for the current incident were included as independent variables.

**Results:**

Multivariable hierarchical logistic regression analyses indicated that males were more likely to be assigned to DS (aOR = 0.40, 95%CI [0.34, 0.46]), and more likely to test positive for use (aOR = 0.43, 95% CI [0.34, 0.54]) than females. As age increased, youth were less likely to be assigned to DS (aOR = 0.91, 95% CI [0.87, 0.94]), with non-significant differences in DS results. Greater number of charges were associated with a higher likelihood of being assigned to DS (aOR = 1.55, 95% CI [1.43, 1.68]). Youth with violent offenses were more likely to be assigned to DS than those with other offense types (property offenses, drug offenses, statutory offenses, disorderly conduct, and all other offenses), but less likely to test positive for use.

**Conclusions:**

Many factors were associated with differences in DS, but these factors were not always associated with differential DS results. Demographic or charge-based decisions may not be appropriate for DS assignment.

**Supplementary Information:**

The online version contains supplementary material available at 10.1186/s40352-024-00273-w.

## Introduction

Substance use among adolescents is associated with numerous negative behavioral and health outcomes, including increased risk for poor school performance and retention, early and unplanned pregnancy, mental health problems, and criminal activity (Bechtold et al., 2015; Chassin et al., 2009). One group of youth who are at particularly high risk for substance use are justice-involved youth (Chassin, 2008; Welty et al., 2016). In fact, one study found that more than a third of a national sample of justice-involved youth had a substance use disorder (SUD), with this rate increasing as justice-involved youth penetrate deeper into the justice system (Wasserman et al., 2010). This prevalence rate for SUD among justice-involved youth is substantially higher than that found among the general population of adolescents in the United States, which is estimated to be 7% (Substance Abuse and Mental Health Services Administration, 2010). The relationship between substance use and justice involvement is complex, particularly given that both variables influence one another (Bennett et al., 2008; Hammersley et al., 1989) and both outcomes are likely the sequalae of a common set of risk factors (Seddon, 2000, 2006). Thus while substance use among justice-involved youth poses similar risk for negative health consequences that have been observed among general population youth, it may be particularly important to understand substance use among justice-involved youth given the increased risk for prolonged justice system involvement among these youth in particular (van der Put et al., 2014; Wiesner et al., 2005).

Given the high rates of substance use among justice-involved youth, justice systems have attempted to monitor use through drug screening (DS) procedures, with some researchers finding that over 70% of youth in contact with community justice agencies are screened at intake for substance use as well as other behavioral health concerns (Wasserman et al., 2021). DS typically involves drug testing (i.e., oral testing, urinalysis, breathalyzer) either at the time of arrest, at juvenile detention, or at intake. Routine, continuous drug testing is also sometimes used with youth on community supervision (i.e., probation).

DS has historically been utilized to both identify and monitor substance use among these youth as well as to identify those youth in need of treatment (Crowe, 1998; Del Carmen & Barnhill, 1999), although it is somewhat unclear how each different jurisdiction across the United States uses drug testing, as system staff and officers sometimes have to rely on their own discretion to decide who is referred for services following DS (Wasserman et al., 2008). Further, there is discretion in deciding who is screened for substance use in the first place, as not every youth who encounters the system is screened (Chassin, 2008; Sickmund & Puzzanchera, 2014). Without the utilization of standardized criteria for DS among justice-involved youth, it is probable that there are biases in deciding which youth receive DS, and subsequently who receives needed substance use services.

To date, research examining whether there are systemic differences in who receives DS among justice-involved youth is lacking, although disparities in screening and referral to treatment exist in at least some jurisdictions across the nation for youth involved in the legal system. For instance, researchers have found that there are no guidelines on screening for substance use disorder, nor what to do with the results of screening (Goldman et al., 2023), and very few juvenile justice systems universally screen and treat youth with SUD (Goldman & Wilson, 2023). This suggests a great deal of variation and discretion in screening for substance use. Additionally, we can look to other fields that illustrate differences in testing based on demographic variables. For instance, in the sexually transmitted infection (STI) literature, some researchers found that high school students referred by school staff for STI testing had significantly higher odds of being male and being multiracial or identifying their race as “other” (Rasberry et al., 2017). Further, in a study examining differential STI testing among symptomatic women, researchers found that providers were less likely to test younger women (ages 14–15) and more likely to test minority women (Black and Latinx) for STIs (Wiehe et al., 2010). While STI/HIV testing is very different than drug testing among adolescents, authority figures (e.g., probation officers) might exhibit similar decision-making processes when it comes to referring youth for drug testing, which some researchers in the STI field suggest is likely due to bias (e.g., Wiehe et al., 2010). Further, studies consistently find that racial/ethnic minority patients (compared to White patients) as well as male patients (compared to female patients) are tested for drug use more frequently in medical settings (e.g., Kon et al., 2004). In one study examining DS in pre-natal medical care, not only were maternal demographic characteristics (i.e., being Black and having a high social and mental health risk factor score) associated with a greater likelihood of prenatal substance use screening, but so were provider attitudes (Kerker et al., 2004).

Social identity theory and stereotypes might offer some explanation of why bias may be observed in these screening disparities across fields. Social identity theory posits that individuals may be more likely to have biases when evaluating someone different than them (e.g., a different gender or race, an outgroup member) than someone who is similar to them (ingroup member; Tajfel & Turner, 1979). This affinity for individuals who are like us could lead to biased decision-making if the youth demographics are discordant with the decision maker’s demographics. Further, regarding stereotypes, some researchers posit that stereotypes of youth of color lead justice personnel (i.e., probation officers) to more negatively assess these youth of color which ultimately impacts decision-making about things like sentencing (Bridges & Steen, 1998).

It is also plausible that if there are differences in DS, system staff may be relying on data that suggests differences in substance use and SUDs among justice-involved youth based on demographic and justice system-related variables. For instance, when considering gender among justice-involved youth, males are more likely to report cannabis and alcohol use compared to females (Braithwaite et al., 2003). Further, age has also been associated with substance use among justice-involved youth, with younger age of substance use onset being associated with greater risk for delinquency and incarceration (Slade et al., 2008). As it relates to race/ethnicity, national data have historically demonstrated that racial and ethnic minority adolescents in the U.S. exhibit lower rates of substance use than their White counterparts (Center for Behavioral Health Statistics and Quality, 2016). Similar disparities in substance use have also been observed among justice-involved youth. For example, studies based on self-report of substance use among justice-involved youth have typically shown that lifetime prevalence rates of any alcohol, cannabis, cocaine, and other drug use are higher among White youth (Braithwaite et al., 2003; Feldstein Ewing et al., 2011).

There have also been differences in substance use found based on other factors related to a youth’s history with the justice system, including number of charges and charge type. Some researchers have found that the number of illicit activities a youth participates in is positively related to subsequent SUDs, as well as the inverse, suggesting a bidirectional relationship between substance use and delinquency (Lalayants & Prince, 2014). Justice-involved youth with SUDs are at an increased risk for escalations in offense severity over time (Hoeve et al., 2013). Further, a high percentage of individuals who are incarcerated in jail for violent crimes report using drugs and/or alcohol at the time of their offense (Snyder et al., 2010; Dorsey et al., 2010). Thus, number of charges and charge type may play a role when deciding who is screened and who might be most likely to test positive for substance use.

However, more research is needed to examine whether there are significant demographic differences within juvenile justice for which youth are assigned to DS and whether these differences are associated with differences in DS results. Understanding differences in court ordered DS by demographic variables may be an important step in understanding and ultimately reducing health disparities among individuals involved in the carceral system (Binswanger et al., 2012). For example, even though White youth broadly report higher rates of substance use, racial and ethnic minority youth report decreased access to both informal and specialty mental health services, a necessity in beginning to address the deleterious effects of substance use in a critical developmental period (Alegria et al., 2011). It is plausible that there may be disparities within the justice-system in which youth of certain demographics or with certain charges are more likely to be assigned to DS based on the discretion of system personnel. This discretionary approach to DS may not effectively leverage the potential of the justice system as a tool to effectively address the deleterious effects of substance use among youth and potentially address disparities in access to specialty substance use treatment.

Further, if biases are present in substance use screening decision-making, this is problematic and could have significant downstream impacts for youth. These decisions can result in youth not being referred or connected to needed services if they are not screened and identified as in need of treatment. Youth not receiving appropriate care can result in an escalation of substance use problems, which has been associated with increased risk for recidivism and escalations in offense severity over time (Chen & Jacobson, 2012; Hoeve et al., 2013).

### Current study

Building on previous research examining drug testing in various care settings, as well as literature examining substance use among justice-involved youth, the current study examined whether demographic variables and charge-related variables were associated with DS assignment (via oral DS tests) and DS results among youth assigned to probation within the juvenile-justice system. Specifically, we sought to examine whether gender, age, race/ethnicity, number of charges for the current incident, or charge type were associated with who was assigned to oral DS and whether their DS result was positive among youth on probation within the juvenile justice system. We hypothesized that rates of testing would be higher for males, younger youth, racial/ethnic minorities, those with a greater number of charges, and those with more severe charge types (e.g., violent offenses). We also hypothesized that rates of positive DS results would be higher for males, older youth, White youth, those with a greater number of charges, and those with more severe charge types. Better understanding the prevalence of DS among justice-involved youth and the results of those screens can potentially elucidate whether biases exist within DS for this population of youth and whether more standardized, data-driven procedures might be warranted.

## Methods

### Sample

**Fig. 1:**
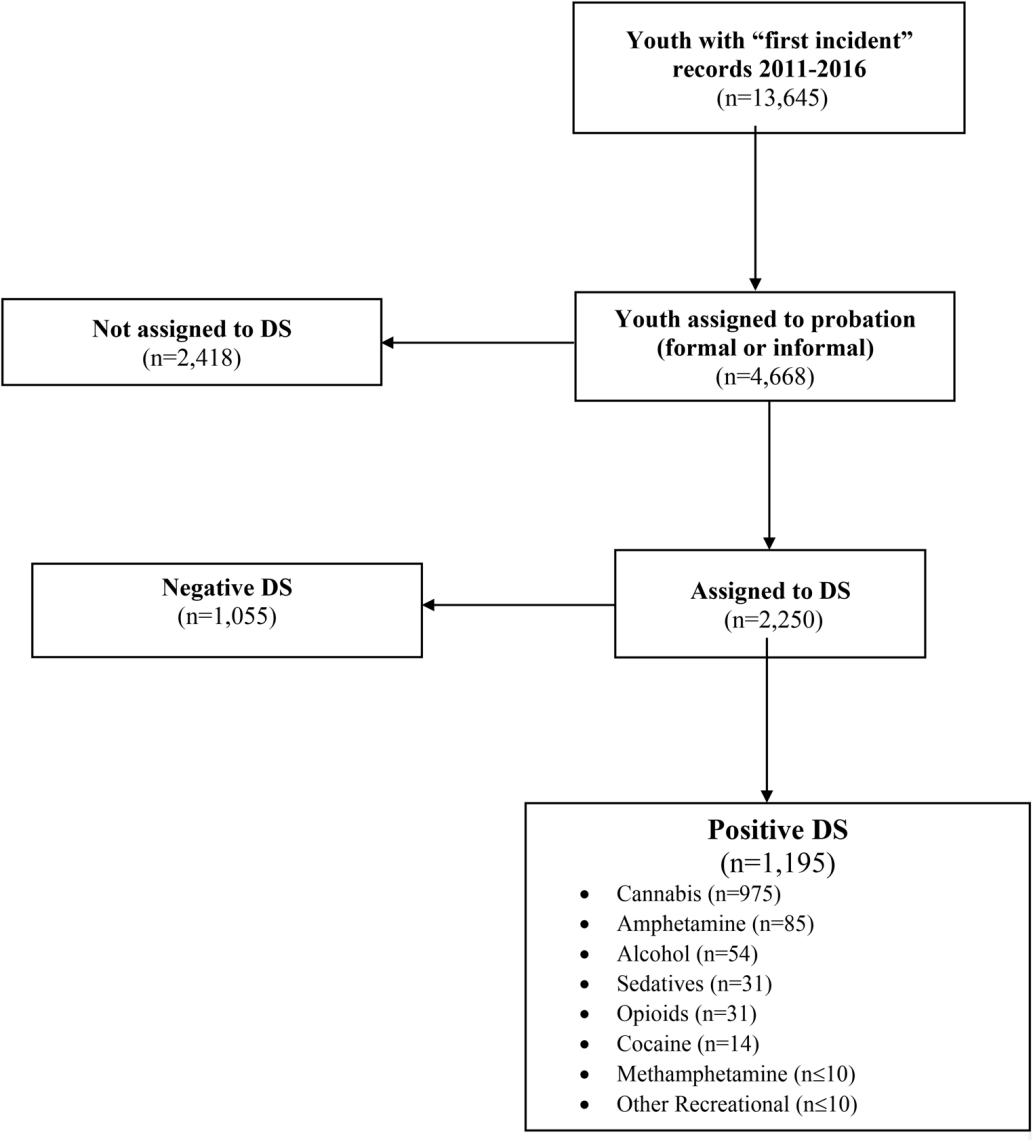
Flow diagram depicting youth who were assigned to DS, and of those, who screened positive for which substances

Following IRB approval, 13,645 youth with records of their first incident (i.e., arrest or justice system referral) in a midwestern urban county’s juvenile justice system between 2011 and 2016 were identified as potential participants for the current study. The following information was gathered from records: race/ethnicity, gender, date of birth, number of charges for the current incident, charge type for the most severe offense in the current incident, probation type, and oral DS results from this first incident. Of note, race/ethnicity and gender data were recorded by justice system personnel and were not necessarily self-reported by youth. Among those with first incident records during the period, 4,668 youth were assigned to a form of probation (formal or informal). A subset of those on probation were assigned to DS and an even smaller subset subsequently tested positive for substance use during this first incident (see Fig. 1 for flow chart). DS results are based on oral drug tests (either 10 or 12-panel Quantisal™ Oral Fluid Collection Devices) which test for a panel of commonly used substances (e.g., cannabis, alcohol, hallucinogens or ecstasy, cocaine, benzodiazepines, methamphetamine, opioids).

### Measures

We examined the associations of race/ethnicity (White, Black, Multi-Racial, Asian, Unknown (missing), Hispanic/Latino, American Indian/Alaska Native, Native Hawaiian), gender (male, female), age, number of charges, and charge type (violent, property, drug, statutory, disorderly, all other) with DS assignment and first DS result for all youth based on electronic court records. Charge types were coded according to the classification system used by the Uniform Crime Reporting Statistics of the U.S.: (1) status offenses (e.g., runaway, curfew violation), (2) disorderly conduct, (3) drug offenses (e.g., dealing, possession), (4) property offenses (e.g., theft, destruction of property), (5) violent offenses (e.g., rape, murder, assault, possession of dangerous weapons), and (6) others (e.g., obstructing emergency medical personnel, unlawful gambling) (United States Department of Justice, Federal Bureau of Investigation, 2009). Dichotomous variables were created to denote DS (yes/no) and DS result (positive/negative).

### Statistical analyses

Data analyses were conducted using the statistical software IBM SPSS Statistics 28. We began by describing the percentage of youth on probation that were assigned to DS by race/ethnicity, gender, age, total number of charges for this incident, and charge type. Some racial/ethnic groups were excluded from analyses due to very low cell size (≤ 5) for both outcomes (see table notes for additional detail). Additionally, there was a very small number of youth with no recorded gender (*n* ≤ 5), this group was also excluded from analyses. These exclusions were made in accordance with sample size suggestions for group difference tests (e.g., McHugh, 2013). Chi-square and t-tests were used to compare those assigned to DS and those not assigned to DS on these demographic and charge-related variables. Further, out of those youth who were assigned to DS, similar analyses were conducted to compare those with positive DS results and those with negative DS results. Finally, for variables with significant initial difference tests, multivariable, hierarchical logistic regression analyses were utilized to examine (1) who was selected for DS, and (2) result of DS. Race/ethnicity (reference group = White), gender (reference group = male), age, number of charges, and charge type (reference group = violent offense) were included as independent variables. Results are presented as adjusted odds ratios with 95% confidence intervals (95% CI). When performing multiple comparisons, there is an increased risk for type 1 errors. Thus, we aimed to limit the false discovery rate while also acknowledging that this is largely an exploratory analysis without much previous research. Thus, we used the Benjamini-Hochberg (BH; Benjamini & Hochberg, 1995) correction procedure to adjust the p-values from the analyses to limit the total false discovery rate to 5%. BH adjusted p-values (*p*_*BH*_) are reported in tables alongside original p-values in tables.

## Results

### Assignment to DS (assigned/not assigned)

Of 4,668 youth on probation following their first incident, 48.2% (*n* = 2,250) were assigned to DS as part of formal or informal probation. Chi-square difference tests initially revealed that there were statistically significant differences between those assigned and not assigned to DS based on race/ethnicity ($\chi$^2 ^= 29.96 (5), *p* < 0.001), gender ($\chi$^2 ^= 290.85 (1), *p* < 0.001), and charge type ($\chi$^2^= 458.33 (5), *p* < 0.001). In addition, t-tests revealed significant differences between those assigned and not assigned to DS based on age (*t* = 2.41(4647.99), *p* = 0.008), and total number of charges for the incident (*t *= -14.93 (3602.62), *p* < 0.001). See Table 1 for full results.

**Table 1:** Descriptive statistics for assignment to DS for youth on probation

Total	Not assigned to DS	Assigned to DS	Total & Difference
	*n* (%) N *=* 2418	*n* (%) N *=* 2250	
Race/ Ethnicity			c^2^ = 29.96 (df = 5)^***^
White	1002 (51%)	961 (49%)	1963
Black	1233 (52.2%)	1127 (47.8%)	2360
Multi-Racial	136 (50.6%)	133 (49.4%)	269
Asian			11
Unknown			26
Hispanic/ Latino			17
Gender			c^2^ = 290.85 (df = 1)^***^
Male	1413 (43.5%)	1834 (56.5%)	3247
Female	1001 (70.6%)	416 (29.4%)	1417
Charge Type			c^2^ = 458.33 (df = 5)^***^
Violent offenses	188 (24%)	591 (76%)	779
Property offenses	673 (51%)	654 (49%)	1327
Drug offenses	355 (44.5%)	442 (55.5%)	797
Statutory offenses	303 (69%)	133 (31%)	436
Disorderly offenses	802 (70%)	346 (30%)	1148
All other offenses	97 (54%)	84 (46%)	181
Total	Mean (SD)	Mean (SD)	
Age at first incident	15.35 (1.77)	15.23 (1.59)	t = 2.41 (df = 4647.99), *p* = 0.008
Total # of charges for this incident	1.45 (0.73)	1.89 (1.24)	t =-14.93 (df = 3602.62)^***^

Note^***^p < 0.001. Percentages are row percentages. Shaded cells represent categories with low cell sizes (*n* ≤ 10) for one or both outcomes (assigned/not assigned) to limit chance of re-identification. Some racial/ethnic groups (American Indian/Alaska Native and Native Hawaiian) were excluded from analyses due to very low cell size (*n* ≤ 5) for one or both outcomes (assigned/not assigned)

These findings were further confirmed based on regression analyses. Regarding gender and age, males were more likely than females to be assigned to DS (aOR = 0.40, 95% CI [0.34, 0.46]) and as age of the youth increased, they were less likely to be assigned to DS (aOR = 0.91, 95% CI [0.87, 0.94]). In regard to charges, youth with property offenses (aOR = 0.42, 95% CI [0.34, 0.51]), drug offenses (aOR = 0.51, 95% CI [0.40, 0.64]), statutory offenses (aOR = 0.25, 95% CI [0.19, 0.33]), disorderly conduct offenses (aOR = 0.21, 95% CI [0.17, 0.26]), and all other offenses (aOR = 0.42, 95% CI [0.29, 0.60]) were less likely than those with violent offenses to be assigned to DS. Additionally, youth with more charges for the current incident (aOR = 1.55, 95% CI [1.43, 1.68]) were also more likely to be assigned to DS. Although there were significant differences in assignment to screening across race/ethnicity for youth identified as Hispanic compared to White youth (aOR = 4.64, 95% CI [1.60, 13.45]), the cell sizes were quite small (less than 1% of the sample) and thus should be interpreted with caution. There were no other significant differences across race/ethnicity. See Table 2 for full results.

**Table 2:** Logistic regression of likelihood of being assigned to DS

	B	SE	aOR (95% CI)	p	BH-adjusted p
Race/ Ethnicity (Ref: White)					
Black	-0.01	0.07	0.99 (0.87, 1.14)	0.914	0.914
Multi-Racial	0.04	0.14	1.04 (0.79,1.38)	0.762	0.914
Asian	-0.49	0.54	0.61 (0.21, 1.78)	0.364	0.914
Unknown	-2.51	1.03	0.08 (0.01, 0.61)	0.015	0.060
Hispanic/ Latino	1.53	0.54	4.64 (1.60, 13.45)	0.005	0.025
Gender (Ref: Male)					
Female	-0.92	0.07	0.40 (0.34, 0.46)	< 0.001	< 0.001
Charge Type (Ref: Violent Offenses)					
Property Offenses	-0.88	0.11	0.42 (0.34, 0.51)	< 0.001	< 0.001
Drug Offenses	-0.68	0.12	0.51 (0.40, 0.64)	< 0.001	< 0.001
Statutory Offenses	-1.38	0.15	0.25 (0.19, 0.33)	< 0.001	< 0.001
Disorderly Conduct	-1.57	0.11	0.21 (0.17, 0.26)	< 0.001	< 0.001
All Other Offenses	-0.87	0.18	0.42 (0.29, 0.60)	< 0.001	< 0.001
Age at first incident	-0.10	0.02	0.91 (0.87, 0.94)	< 0.001	< 0.001
Total # of charges for this incident	0.44	0.04	1.55 (1.43, 1.68)	< 0.001	< 0.001

*Note* Regression analyses only conducted on variables where initial difference tests were significant. aOR = adjusted odds ratio. All variables shown in table were included in the model

### DS result (positive/negative)

Further, 25.60% of the youth on probation following their first incident (*n* = 1,195) had a positive DS result. Chi-square difference tests initially revealed that there were significant differences between those with positive DS results and those with negative DS results based on gender ($\chi$^2 ^= 49.13 (1), *p* < 0.001) and charge type ($\chi$^2 ^= 81.24 (5), *p* < 0.001). There were no significant differences for the other demographic and charge-related variables. See Table 3 for full results. Males were more likely than females to test positive (aOR = 0.43, 95% CI [0.34, 0.54]). Additionally, youth with property offenses (aOR = 2.63, 95% CI [2.08, 3.33]), drug offenses (aOR = 2.09, 95% CI [1.60, 2.71]), statutory offenses (aOR = 3.06, 95% CI [2.03, 4.60]), disorderly conduct offenses (aOR = 2.15, 95% CI [1.62, 2.87]), and all other offenses (aOR = 5.36, 95% CI [3.16, 9.11]) were more likely than those with violent offenses to test positive. See Table 4 for full results.

**Table 3:** Descriptive statistics for DS result for youth on probation assigned to DS

Total	Negative DS	Positive DS	Total & Difference
	*n* (%) N = 1055	*n* (%) N = 1195	
Race/Ethnicity			c^2^ = 4.42 (df = 2), *p* = 0.108
White	470 (48.9%)	491 (51.1%)	961
Black	501 (44.5%)	626 (55.5%)	1127
Multi-Racial	65 (48.9%)	68 (51.1%)	133
Gender			c^2^ = 49.13 (df = 1)^***^
Male	795 (43.3%)	1039 (56.7%)	1834
Female	260 (62.5%)	156 (37.5%)	416
Charge Type			c^2^ = 81.24 (df = 5)^***^
Violent offenses	360 (60.9%)	231 (39.1%)	591
Property offenses	250 (38.2%)	404 (61.8%)	654
Drug offenses	197 (44.6%)	245 (55.4%)	442
Statutory offenses	58 (43.6%)	75 (56.4%)	133
Disorderly offenses	167 (48.3%)	179 (51.7%)	346
All other offenses	23 (27.4%)	61 (72.6%)	84
Total	Mean (SD)	Mean (SD)	
Age at first incident	15.19 (1.58)	15.27 (1.60)	t = -1.26, (df = 2212.87), *p* = 0.103
Total # of charges for this incident	1.86 (1.09)	1.92 (1.35)	t = -1.20, (df = 2225.53), *p* = 0.115

Note^***^p < 0.001. Percentages are row percentages. Some racial/ethnic groups (American Indian/Alaska Native, Native Hawaiian, Unknown, Asian, Hispanic/Latino) were excluded from analyses due to very low cell size (*n* ≤ 5) for one or both outcomes (assigned/not assigned)

**Table 4:** Logistic regression of likelihood of positive UDS

	B	SE	aOR (95% CI)	p	BH-adjusted p
Gender (Ref: Male)					
Female	-0.85	0.12	0.43 (0.34, 0.54)	< 0.001	< 0.001
Charge Type (Ref: Violent Offenses)					
Property Offenses	0.97	0.12	2.63 (2.08, 3.33)	< 0.001	< 0.001
Drug Offenses	0.74	0.13	2.09 (1.60, 2.71)	< 0.001	< 0.001
Statutory Offenses	1.12	0.21	3.06 (2.03, 4.60)	< 0.001	< 0.001
Disorderly Conduct	0.77	0.15	2.15 (1.62, 2.87)	< 0.001	< 0.001
All Other Offenses	1.68	0.27	5.36 (3.16, 9.11)	< 0.001	< 0.001

*Note* Regression analyses only conducted on variables where initial difference tests were significant. aOR = adjusted odds ratio. All variables shown in table were included in the model

## Discussion

This study sought to examine demographic and charge-related factors associated with DS and the results of those screens following a youth’s first incident with the juvenile justice system resulting in probation. The findings were mostly in line with our hypotheses, which we discuss in more detail below.

To date, there are no national guidelines for standardizing DS protocols within juvenile justice settings. It is unknown what decision-making processes were used by justice system personnel in this study to determine who was screened. For instance, an individual probation officer, probation officer supervisor, or judge could have determined that the youth should receive DS. Future research should elucidate the roles of specific court actors in making DS decisions. Although the nature of the current data does not allow for understanding the rationale behind screening selection, it is plausible that decisions regarding screening could be based on personnel’s knowledge about rates of substance use among youth in this community.

While knowledge of one’s community is useful for justice personnel, there are important considerations regarding the intersectionality of demographic variables, development, and context that influence risk for substance use among justice-involved youth that might not be apparent when only examining community-level substance use statistics. For example, in one longitudinal study of substance use, researchers found that females were using substances more frequently than males early in adolescence; however, beginning in mid-adolescence, males were using substances more frequently (Chen & Jacobson, 2012). Thus, perhaps a number of other factors predict gender differences in substance use behaviors and outcomes, and we might be missing some of this information if we are arbitrarily administering more DS to justice-involved males than to females. This is supported by research suggesting that there are differences in risk factors for substance use among justice-involved females compared to males, including contextual risk factors such as parental monitoring, peer substance use, peer violent behavior, and personal risk factors, such as impulsivity, internalizing behaviors, risk-taking behavior (Herrera & Boxer, 2019). Thus, perhaps along with considering potential gender differences in substance use, contextual and personal risk factors impacting substance use among justice-involved youth should also be considered. These risk factors might be important variables to consider if systems are to adopt more standardized substance use screening procedures.

There was also some evidence that youth were assigned to DS based on charges (both number of charges and severity). Youth with a greater number of charges and with violent charges were more likely to be assigned to DS. Goldstein’s conceptual framework suggests that substance use leads to violent behavior (Goldstein, 1985). This framework outlines the ways psychopharmacological, economic compulsion, and systemic factors link substance use and violence. These factors can also help to explain how substance use might be related to a wide range of criminal/criminalized behaviors. Further, studies conducted among nationally representative samples suggest that substance use is predictive of type and severity of arrest charges (Kopak & Hoffmann, 2014). Thus, it follows conceptually that individuals with more charges and those with more violent charges may be assigned to DS by probation. In contrast, our findings suggest that individuals with non-violent charges (including property offenses, drug offenses, statutory offenses, disorderly conduct offenses, and all other offenses) were more likely to have a positive DS result compared to those individuals with violent offenses. So, while we might expect that substance use and violent behavior are linked, other charge types might be even more strongly associated with substance use among justice-involved youth. Kopak and Hoffman (2014) found that drug dependence increased the probability of being charged with a non-violent crime, and cited previous work asserting that acquisitive crimes comprise a consequential portion of offending among individuals engaged in substance use in the criminal justice system (Rogne Gjeruldsen et al., 2004). Thus, again, while one might think individuals with violent offenses use substances more frequently, our study, in concert with previous work, suggests that individuals with non-violent offenses are also likely to engage in substance use. In turn, standardization of DS procedures might take some of the guesswork out of who is most at risk and who should be screened.

We did not find differences in assignment to screening based on race/ethnicity which is inconsistent with our hypotheses. Despite lack of findings, additional research is needed to further understand potential impact of race/ethnicity on system decision-making with respect to DS given evidence for disproportionate minority contact with the justice system. Regarding substance use, findings for disproportionate minority contact are suggested to be related to the common stereotype of the “dangerous drug offender” that is more often applied to racial/ethnic minorities than Whites (Leiber et al., 2017).

Given the results of the current study, questions remain regarding how justice systems/personnel are expected to make decisions about who should be tested for substance use. Justice system decision-making regarding testing is important as, in the current study, certain demographic groups and youth with specific types of charges were tested more frequently than others. DS can be used by juvenile justice system staff as a means of determining case disposition and service recommendations or for disciplinary monitoring (Belenko et al., 2017; Dir et al., 2021). Thus, biases in screening could lead to biases in adjudication decisions and system penetration.

Reviewing substance use testing policies in other settings may be useful as we work to understand and develop appropriate standardization procedures in DS among justice-involved youth. In some medical settings, providers and system level organizations have called for universal DS policies. For example, in 2015 the American College of Obstetricians and Gynecologists supported universal substance use screening as a part of prenatal care for all pregnant individuals followed by appropriate referral and intervention (Mascola et al., 2017). While research from other fields suggests that standardized screening and assessment for substance use could be an appropriate approach, more work is needed exploring whether implementing strategies for substance use screening standardization in juvenile justice settings leads to more equity in adjudication decisions and system penetration among demographic groups.

Despite these ongoing practical and ethical challenges to administering DS, some justice systems have piloted systems to standardize DS administration. For example, Hawaii’s Opportunity Probation with Enforcement (HOPE) program relies on standardized DS coupled with “swift and certain” sanctions (Cadwallader, 2017). This program mandates a certain number of random DS be administered to all individuals who are part of this program and there are a set range of consequences depending on the result of that screening (Cadwallader, 2017). This eliminates the probation officer’s need to utilize their own discretion about who should be administered DS and further creates standardized responses to positive DS results.

However, while standardized assessment could be helpful, it will be important to consider the cost and feasibility of administering DS in a standardized manner, as well as the potential stigma associated with receiving DS for justice-involved youth. Uncritically testing all justice-involved youth may prove to be even more burdensome, costly, and overall infeasible, eventually leading to worse intervention outcomes. Many justice systems are strained by limited resources and selecting only youth who are most likely to engage in substance use would allow for more targeted use of resources for those who would be most likely to benefit from referral to services. Leveraging data regarding variables related to DS and subsequent substance use or non-use could inform such a targeted approach. This, in turn, could more effectively maximize the “return on investment” for DS in that it would allow justice systems to integrate a data-driven approach with broader knowledge of developmental influences on substance use in referring youth for DS rather than relying on discretion alone. There is also a substantial body of research illustrating the negative impact of inappropriately restrictive court supervision, particularly while youth are on probation. For instance, some argue that the more requirements that youth are assigned while on probation (e.g., electronic monitoring, curfew conditions, DS, education programs, completion of community service, etc.), the more difficult it is for youth to complete all requirements, leading to probation violations (Dir et al., 2021) and unnecessarily prolonged system involvement (NeMoyer et al., 2014). Further, researchers suggest that justice systems should assess substance use screening instruments for accuracy, length, cost and window of detection (Knight et al., 2003); screening youth who do not need to be screened may result in unnecessary strain on justice system and youth resources. Thus, perhaps discretionary DS could be advantageous in some settings.

### Limitations and future directions

This study has many important strengths, for example documenting the demographic factors associated with DS and outcomes among justice-involved youth, but it is not without limitations. First, the results presented in this paper are only for first incident records. There may be additional differences in assignment to DS and positivity of those tests as an individual persists in the justice system or recidivates. More research is needed to examine this. Second, oral DS records only offer a small window into actual substance use patterns. There are likely many youth who had negative screening results with a history of substance use as well as youth with positive DS results who used other unidentified substances. Thus, the results regarding DS positivity should be interpreted with that information in mind and in relation to the results illustrating who is most likely to be assigned to DS. Third, we had a limited sample size to examine differences in outcomes based on race/ethnicity and acknowledge that it can be difficult to draw conclusions based on such small cell sizes. Future research should aim to continue looking at differences in DS assignment and DS result by race/ethnicity with larger sample sizes. Fourth, we did not have information regarding justice system decision-making regarding DS. This information is pertinent from both a research and system-level perspective and highlights a need for a thorough documentation of the personnel involved in making substance use testing decisions, as well as their rationale for their decisions. Perhaps a qualitative study with system personnel would also help to illuminate this process. Additional research examining predictors of assignment to DS and DS positivity among justice-involved youth is warranted.

## Conclusions

Substance use among adolescents is a public health problem, particularly among justice-involved youth. The justice system is plagued by disparities based on race/ethnicity, gender, and several other factors. In this study, we found that males were more likely to be assigned to DS and more likely to test positive for substance use than females. Individuals with more charges were more likely to be assigned to DS, whereas individuals with violent offenses were more likely to be assigned to DS than those with any other offense type, but less likely to test positive for substance use. This is the first study, to our knowledge, to examine demographic factors associated with assignment to DS among justice-involved youth. The findings presented have significant public health implications relating to potential biases influencing decision-making among justice system personnel. The results underscore the need for additional research to understand the justice system processes of identifying and assigning youth to DS to optimize and standardize this decision-making process in service of improving substance use outcomes and potentially reducing substance use treatment disparities.

### Electronic supplementary material

Below is the link to the electronic supplementary material.

Supplementary Material 1

## Data Availability

The datasets analyzed during the current study are not publicly available to protect the privacy of minors with legal system involvement.
