# Supplementary material for: Who gets screened and who tests positive? Drug screening among justice-involved youth in a midwestern urban county
Source: Health Justice. 2024 Apr 5;12:13. doi: 10.1186/s40352-024-00273-w (PMC10997690; doi:10.1186/s40352-024-00273-w)
Supplement: Supplementary file 1 — Supplementary Material 1 [file 40352_2024_273_MOESM1_ESM.docx]

Dear Dr. Gideon,

Thank you for providing the opportunity to revise and resubmit our manuscript, "Who gets screened and who tests positive? Drug screening among justice-involved youth in a midwestern urban county" (Manuscript #: HEJU-D-22-00080), for review to *Health & Justice.* We greatly appreciate the thoroughness of the reviews provided, which we believe has resulted in a stronger paper. This letter accompanies the resubmission. In this letter, we summarize each comment made by reviewers, and describe the changes we made to the paper in response. Additionally, we have highlighted changes made in the manuscript by using track changes. Thank you again for the opportunity to resubmit.

**Reviewer 1 Comments:**

1. The analytic method should be clarified in the abstract.

**Response:** Information about the analytic method has been added to the abstract.

2. Please give an indication of the general region in which the study took place in the title and abstract.

**Response:** Region where the data for this study was collected has been added to the title and abstract.

3. Provide confidence limits rather than p-values in the abstract results.

**Response:** Confidence limits are now provided in abstract results.

4. **4.** Ensure that the findings described in the conclusions (e.g. on violent offenses) are reflected in the results of the abstract.

**Response:** Results for Charge Type variable have been added to the abstract.

5. The sentence in the first paragraph that indicates justice-involved youth are at risk for substance is somewhat confusing - isn't the cause and effect in the other direction (youth who use substances are at risk for justice involvement)?

**Response:** We appreciate this comment and the opportunity to clarify this in the manuscript. Researchers suggest that this process is complex and can be bidirectional where substance use and justice involvement both impact the other and additionally, drug use and justice involvement likely share common sets of precipitating factors and therefore cooccur (Chassin et al., 2008; Hammersley et al., 1989; Bennet & Holloway, 2009; Seddon, 2000). The language in the first paragraph has been edited to reflect this complexity.

6. Can the authors indicate early in the second paragraph if screening is used in youth to decide who gets services and what kind?

**Response:** Information has been added to the second paragraph regarding how drug screens are used. However, we also note that it is unclear how each jurisdiction chooses to use drug testing.

7. What kind of screening is commonly used in youth? Is it drug-specific? When does it get used and how often? Please clarify for an audience who might not be familiar with such procedures in the youth justice system.

**Response:** Drug screening is common practice in juvenile justice systems (Wasserman et al., 2021); most youth who interact with the justice system undergo drug testing (i.e., oral testing, urinalysis, breathalyzer) either at the time of arrest, at juvenile detention, or at intake. Routine, continuous drug testing is also sometimes used with youth on community supervision (i.e., probation); however, these practices vary across jurisdictions. The current sample includes only youth on probation, and in this jurisdiction, it is common practice but not uniform that all youth engage in routine drug testing. In the jurisdiction which the sample is based on, drug testing is not drug-specific; rather, oral drug testing kits (either 10 or 12-panel kits) test for a panel of commonly used substances. There is heterogeneity in how drug testing is utilized across different jurisdictions, and even within jurisdictions, there may be differences in the frequency of drug testing. We hope this study sheds light on some of the inconsistencies in drug testing and hope to understand patterns in decision-making regarding drug testing. Information about common drug screening practices as well as the methods of drug screening utilized in the data analyzed in the current study has been added to the manuscript in the introduction (pgs. 2-3) and methods (pg. 8).

8. Consider the term "sexually transmitted infections" instead of "sexually transmitted diseases." Most would classify these as infections rather than diseases.

**Response:** We appreciate this comment and have edited language to discuss sexually transmitted infections (STI).

9. Minor: page 3 line 66, data should be plural, as in "data have historically"

**Response:** We have reviewed the manuscript and edited for typographical and grammatical errors, including the specific error mentioned here (pg. 5, line 204).

10. Minor: page 4 line 80, rather than the term "substances" clarify if this drugs or drugs and alcohol. The current manuscript appears to be about drugs; therefore, consider citing the specific statistics that relate to drugs rather than drugs and alcohol here.

**Response:** DS described in the current manuscript included both drugs and alcohol. This literature review section has been edited to reflect the information is about both drugs and alcohol (pg. 5, line 218).

11. Page 4, line 83: Would not speculate on the whole body of literature here - would just indicate the authors perceive this as a scientific gap.

**Response:** This line has been edited in line with this suggestion (pg. 6, line 227).

12. Page 4, line 85: The early use of the term bias to describe difference indicates that the authors seem to favor the theory that DS is predominantly negative, as opposed to beneficial, for youth. Please clarify when and how the authors would consider a difference a bias vs. a difference or use a more neutral term here and discuss the implications of the findings for biases vs. differences in the discussion.
    **Response:** The term “bias” has been removed from this section of text and replaced by “differences in court ordered DS by demographic variables (pg. 6, line 229).
13. Please provide some context into how DS is used in the setting studied in the methods.

**Response:** We are unable to describe why or how DS is used in this setting, in that we are uncertain as to decision-making regarding who gets tested, how often, and how drug testing may impact court decision-making. This is part of the aim of the study is to start to explore patterns in the practice of DS. We do know that DS is common practice in the juvenile justice system; however, practices vary across jurisdictions. The current sample includes only youth on probation, and drug testing is often used as part of court monitoring for youth on probation. We do know that youth in our sample were arrested, placed on probation and ordered to receive drug screening. We cannot determine how the results were used. We have, however, provided some information about the use of DS generally to the introduction (pg. 3).

14. Describe the oral DS tests if possible in methods - what drugs did they detect? How are they administered/by whom? Since the authors examine positive test rates, it is important to understand the meaning of these tests.

**Response:** Known additional information about DS has been added to the methods (pg. 8).

15. When adjusted odds ratios and confidence limits are provided, the p-values are not necessary.

**Response:** The p-values have been removed from the text of the results section.

16. MINOR: Page 11, line 235 and line 243 appear to be missing a word.

**Response:** We have reviewed the manuscript and edited for typographical and grammatical errors, including the specific error mentioned here.

17. The paragraph spanning lines 223-251 is too long and dense.

**Response:** We have reviewed the manuscript and edited for typographical and grammatical errors, including the specific error mentioned here.

18. Again, clarify if substance use leading to violence is about drugs or alcohol (page 12, line 255). If about alcohol, this point is not important to the research discussed in this manuscript and can be deleted.

**Response:** DS described in the current manuscript included both drugs and alcohol and thus “substance use” language is retained in this section of the paper (pg. 12, line 535).

19. Page 12, consider alternative terms to drug users to minimize stigma.

**Response:** We appreciate this reviewer's attention to the use of potentially stigmatizing terminology in this manuscript. We have reviewed the article for other instances of stigmatizing language, editing where necessary, including in the instance highlighted in this comment.

20. It is not important to highlight that this is the first study to do x and y in the conclusion.

**Response:** This language has been removed from the conclusion section.

21. Overall, the discussion could be improved. It could be more concise, and some concepts described in the introduction may not need to be repeated. For instance, the pros and the cons of screening could be grouped in the introduction (including the iatrogenic potential), and the lack of guidelines could also be highlighted in the introduction. The discussion could then be focused on how this study compares/contrasts to the other literature/theory, which the findings represent biases or differences, or just a lack of standardization, and what next steps are needed in research and practice. This is not meant to be a prescriptive suggestion but just to highlight that the organization was not as clear as it could have been, making it more difficult to read.
    **Response:** We appreciate these suggestions and have worked to better organize the introduction and conclusion sections, as well as reducing some of the information presented in the discussion section.

**Reviewer 2 Comments:**

1. To strengthen the paper's contribution to the literature, the authors should more directly address reasons why differences in drug screening are expected. Specifically, the introductory sections of the paper would benefit from some theoretical explanation and exploration for why differences in drug screening are likely.
   **Response:** We agree that it may be helpful to orient the reader to additional reasons we might expect differences in DS. Some possible theoretical explanations for differences in DS have been added to the introduction (pg. 4).
2. Is drug screening discretionary across the country? Was a review of state legislation and departments of juvenile justice policy/procedure conducted to confirm this? Just as in the adult system, there are some circumstances why drug screening may be mandatory. The author(s) should better explain and explore how discretionary drug screens are and whether the level of discretion is similar across jurisdictions outside of the study site.
   **Response:** We did not review state legislation nor state practice guidelines to determine drug screening discretion. We do know that disparity in screening and referral to treatment exist in at least some jurisdictions across the nation for youth involved in the legal system. For instance, researchers have found that there are no guidelines on screening for substance use disorder, nor what to do with the results of screening (Goldman et al., 2023), and very few juvenile justice systems universally screen and treat youth with SUD (Goldman & Wilson, 2023), which suggests a great deal of variation and discretion in screening for substance use. This information has been added to the introduction of the paper (pg. 3).
3. A discussion of why drug screening is discretionary would also be helpful. Are there concerns with testing everyone (high rate of false positives or negatives), lack of staffing and resources, concerns about privacy, etc.?

**Response:** Some information about the potential advantage of discretionary testing has been added to the discussion (pg. 16).
